# Quasi-Vertical GaN-Based Trench Gate MOSFETs Treated with Fluorine-Plasma

**DOI:** 10.3390/mi17091019

**Published:** 2026-08-27

**Authors:** Peng Zhang, Dong-Yang Li, Jia-Rui Xu, Qing-Yuan Chang, Bin Hou, He Xi, Xiao-Hua Ma

**Affiliations:** Key Laboratory of Wide Band Gap Semiconductor Materials and Devices, Faculty of Integrated Circuit, Xidian University, Xi’an 710071, China; pengzhang@xidian.edu.cn (P.Z.); 24251215659@stu.xidian.edu.cn (D.-Y.L.);

**Keywords:** quasi-vertical GaN, trench gate, MOSFETs, F-plasma-treated, breakdown voltage

## Abstract

In this paper, a quasi-vertical GaN-based trench gate MOSFETs treated with F-plasma is proposed. The F-plasma-treated devices exhibit a breakdown voltage (V_br_) increased from 170 V to 275 V compared to the untreated devices. TCAD simulations reveal that F-plasma treatment significantly reduces the peak electric field of the device, lowering the peak electric field from 18.7 MV/cm to 4.08 MV/cm (at a drain voltage of 900 V). For the F-plasma-treated device with a 4 μm gate trench dimension, the maximum saturation current is 2258.2 A/cm^2^, and the specific on-resistance (R_on,sp_) is 7.2 mΩ·cm^2^. Stress testing results show that both the treated and untreated devices have their threshold voltage affected by interface charges and traps, with no additional impact of the F-plasma treatment on the threshold voltage stability of the device.

## 1. Introduction

GaN possesses excellent electrical properties, including a wide bandgap, a high critical breakdown electric field, and a high saturation velocity, which enable the resulting devices to withstand higher breakdown voltages. Moreover, at the same voltage rating, GaN-based devices exhibit lower on-resistance, rendering them highly promising for high-voltage and high-power applications in power electronics. Vertical GaN transistors offer prominent advantages: notably, they can achieve high breakdown voltages by increasing the drift layer thickness without expanding the device footprint. Among them, trench-structured GaN MOSFETs are particularly attractive due to their simpler fabrication process and relatively higher threshold voltage (Vth) compared with other types of vertical transistors [1]. In contrast to lateral HEMT devices, vertical GaN MOSFETs adopt a vertical topology, which allows high-voltage chips to be achieved by increasing the thickness of the drift region without sacrificing device area. Moreover, vertical GaN MOSFETs exhibit lower sensitivity to surface trap states relative to their lateral counterparts, effectively mitigating dynamic on-resistance degradation and suppressing current collapse. Given that commercially available GaN substrates are limited to 4-inch wafers, quasi-vertical GaN MOSFETs fabricated on Si substrates feature lower cost and simpler processing, providing a cost-effective alternative for the development of vertical GaN MOSFET structures. However, one critical challenge in GaN trench MOSFET design is the severe electric field concentration at the trench bottom under off-state conditions, which can significantly degrade the breakdown voltage [1,2,3,4,5,6,7,8,9,10]. To reduce the peak electric field, Dong Ji et al. fabricated vertical GaN-based MOSFETs with gate-source dual field plates, achieving a low specific on-resistance (R_on,sp_) of 2.2 mΩ·cm^2^ and a breakdown voltage exceeding 1400 V [2]. Zhu et al. adopted a thick gate dielectric at the trench bottom to suppress the peak electric field, raising the breakdown voltage fivefold from 95 V to 485 V while preserving a low R_on,sp_ of 0.95 mΩ·cm^2^ [6]. Alternatively, shielded gate architectures have been proposed to mitigate the peak electric field, but at the cost of increased fabrication complexity [11]. For lateral HEMTs and power diodes, F-plasma treatment has been widely validated as an effective electric field management strategy with a relatively simple process flow [12,13,14,15]. Nevertheless, to date, the application of F-plasma treatment to quasi-vertical GaN MOSFETs remains unreported. The principle of F-plasma treatment lies in the introduction of fixed negative charges, which repel electrons and form a depletion region. The underlying mechanism of F-plasma treatment relies on the incorporation of fixed negative charges, which repel majority electrons and induce a local depletion region. This effect serves to spread the electric field profile and reduce the peak field magnitude. Accordingly, this strategy can be extended to quasi-vertical GaN MOSFETs to achieve enhanced breakdown performance.

In this paper, we propose for the first time a gate-on-both-sides combined with F-plasma treatment to reduce the peak electric field strength at the gate edge of quasi-vertical GaN-Based trench gate MOSFETs. The results show that F-plasma treatment significantly reduces the peak electric field of the device, leading to an increase in the breakdown voltage from 170 V to 275 V.

## 2. Materials and Methods

**Figure 1:**
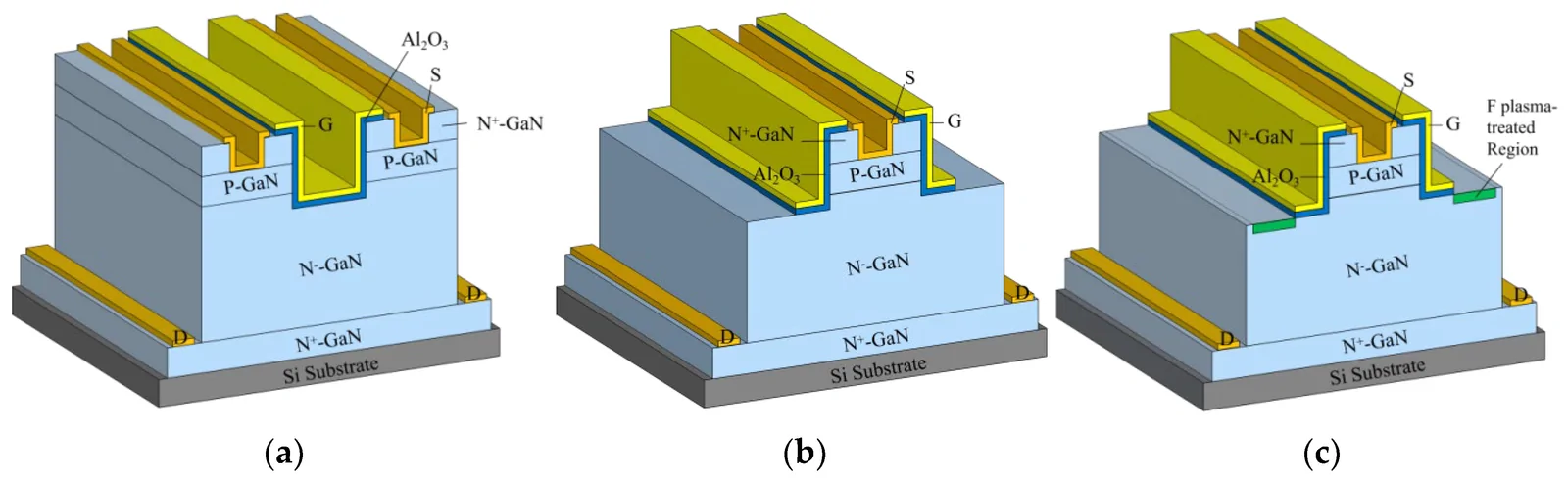
(**a**) Structure diagrams of traditional device A. (**b**) Structure diagrams of traditional device B. (**c**) Structure diagrams of F-plasma-treated quasi-vertical GaN-based trench gate MOSFETs.

The schematic of the F-plasma-treated quasi-vertical GaN-based trench gate MOSFETs is shown in Figure 1. The traditional device A adopts a gate-in-the-middle structure without F-plasma treatment, while the traditional device B employs a gate-on-both-sides structure, also without F-plasma treatment. Since the gate-in-the-middle structure prevents F-plasma treatment due to the shielding effect of the gate metal, the gate-on-both-sides structure was selected for F-plasma treatment, as shown in Figure 1c. From bottom to top, the main structural parameters of the device are as follows: a 1 µm N^+^-GaN layer [Si:3 × 10^18^ cm^−3^], a 4 µm N^−^-GaN drift layer [Si:1 × 10^16^ cm^−3^], a 350 nm P-GaN layer [Mg:1 × 10^19^ cm^−3^], and a 220 nm N^+^-GaN layer [Si:1 × 10^19^ cm^−3^]. The device manufacturing process begins with the etching of a 700 nm deep trench using a Cl_2_/BCl_3_ gas mixture in a 15 W inductively coupled plasma (ICP) etching system. After the trench is formed, a 25% TMAH solution is used for a 1 h water bath treatment at 80 °C. The device is then annealed at 700 °C for 10 min to activate the buried P-GaN. Subsequently, a 50 nm thick Al_2_O_3_ layer is deposited as the gate dielectric using atomic layer deposition (ALD). The source and drain are made with Ti/Al/Ni/Au as the ohmic metal, while Ni/Au (45 nm/400 nm) is used as the gate metal. Finally, F-plasma treatment is performed to complete the device fabrication. The F-plasma treatment process involves 150 s of CF4 plasma treatment using the ICP system, with a CF_4_ gas flow rate of 45 sccm, an ICP power of 180 W, and a bias power of 55 W during the treatment.

## 3. Results

**Figure 2:**
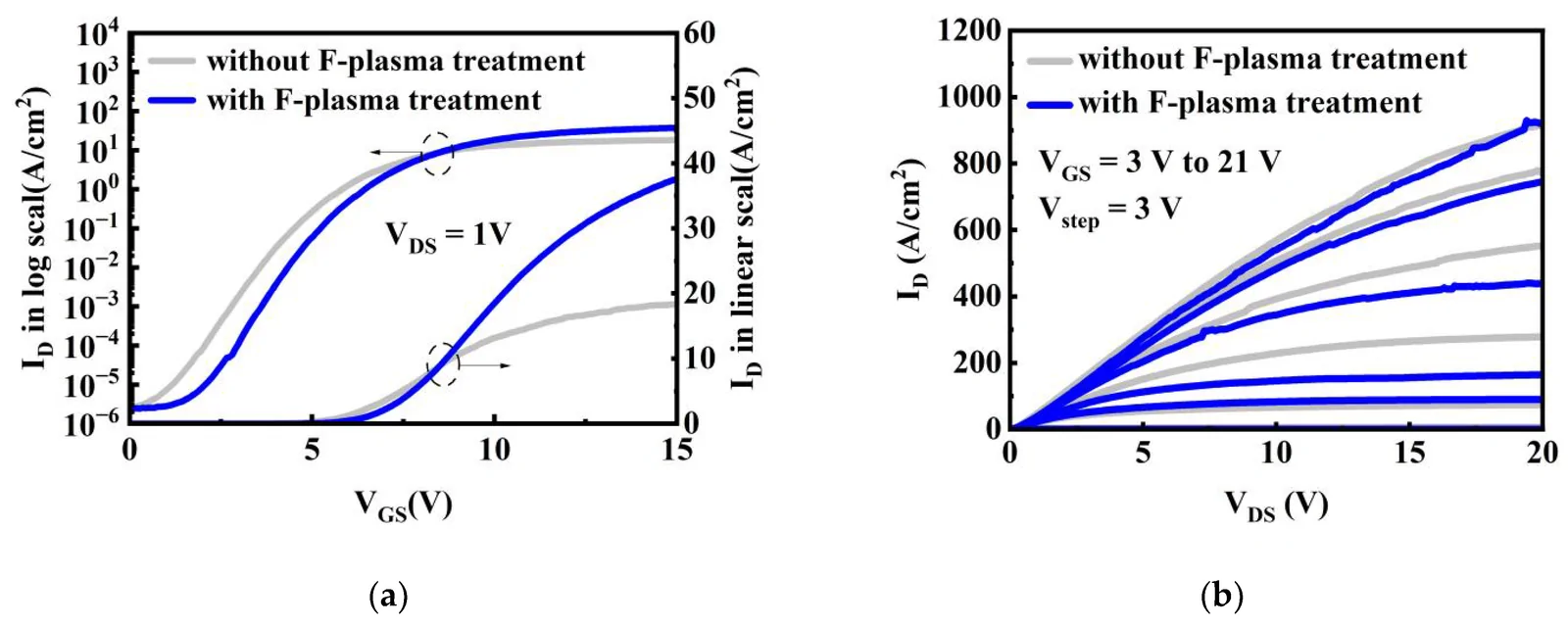

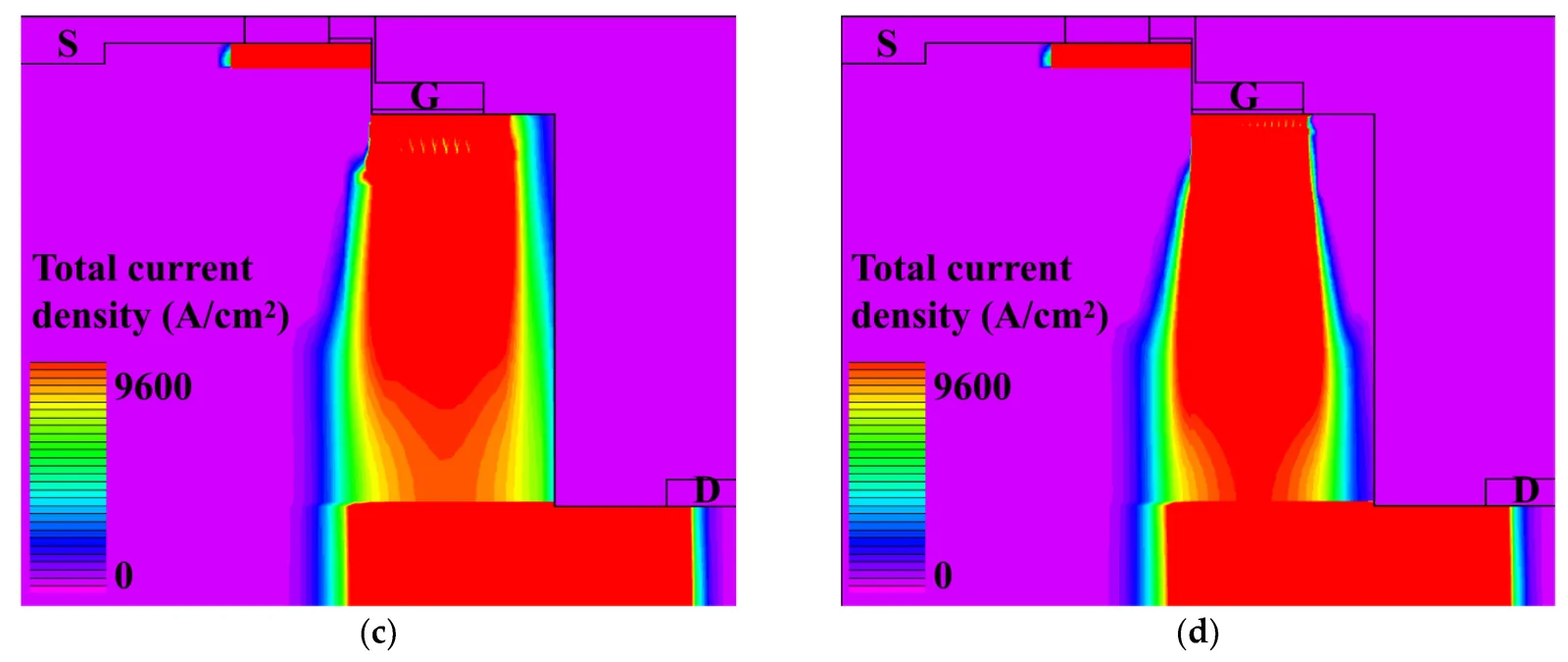
Comparison of (**a**) transmission and (**b**) output characteristics of F-plasma-treated and untreated devices based on simulation. Current density distributions of the device (**c**) without and (**d**) with F-plasma treatment at V_GS_ = 20 V and V_DS_ = 20 V based on simulation.

Figure 2a,b compare the transfer and output characteristics of the as-fabricated quasi-vertical GaN-based trench-gate MOSFETs with and without F-plasma treatment. The test device features a total gate trench dimension of 8 µm × 135 µm. As depicted in Figure 2a, the threshold voltages of the untreated and F-plasma-treated samples are measured as 5.8 V and 6.4 V, respectively. Both devices achieve an on/off current ratio (I_on_/I_off_) on the order of 10^5^. As shown in Figure 2b, the saturation current of the F-plasma-treated quasi-vertical GaN MOSFETs is lower than that of the untreated counterparts. Nevertheless, at a high gate bias of 21 V, the gap in saturation current becomes negligible, with both devices delivering a current density of approximately 917 A/cm^2^. Figure 2c,d illustrate the on-state current distribution in the quasi-vertical GaN-based trench-gate MOSFETs with and without F-plasma treatment. The results indicate that the fixed negative charges introduced by F-plasma treatment repel electrons and induce a local depletion region, which accounts for the reduced saturation current. With a scaled-down gate trench size, the device yields a maximum saturation current of 2258.2 A/cm^2^ and a specific on-resistance (R_on,sp_) of 7.2 mΩ·cm^2^.

**Figure 3:**
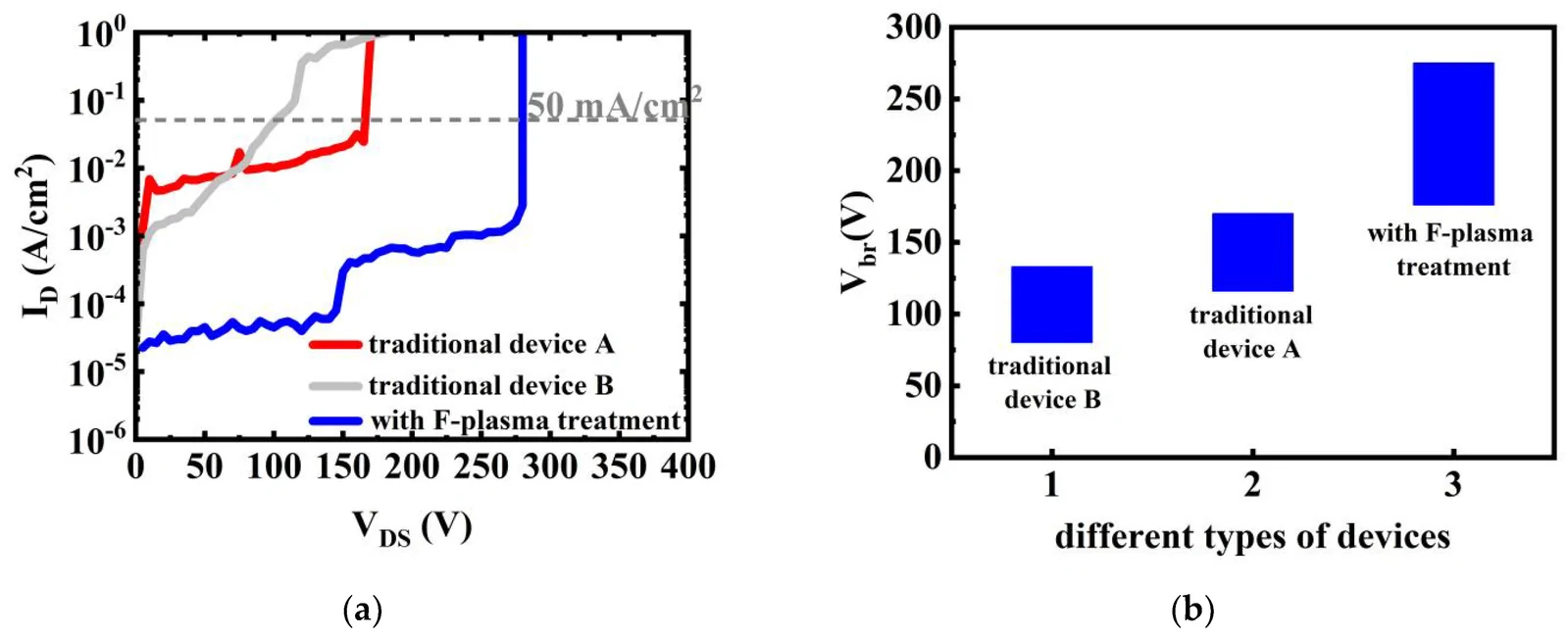
(**a**) Off-state characteristic curve of the quasi-vertical MOSFET device. (**b**) Voltage distribution map of the tested device (V_GS_ = 0 V).

**Figure 4:**
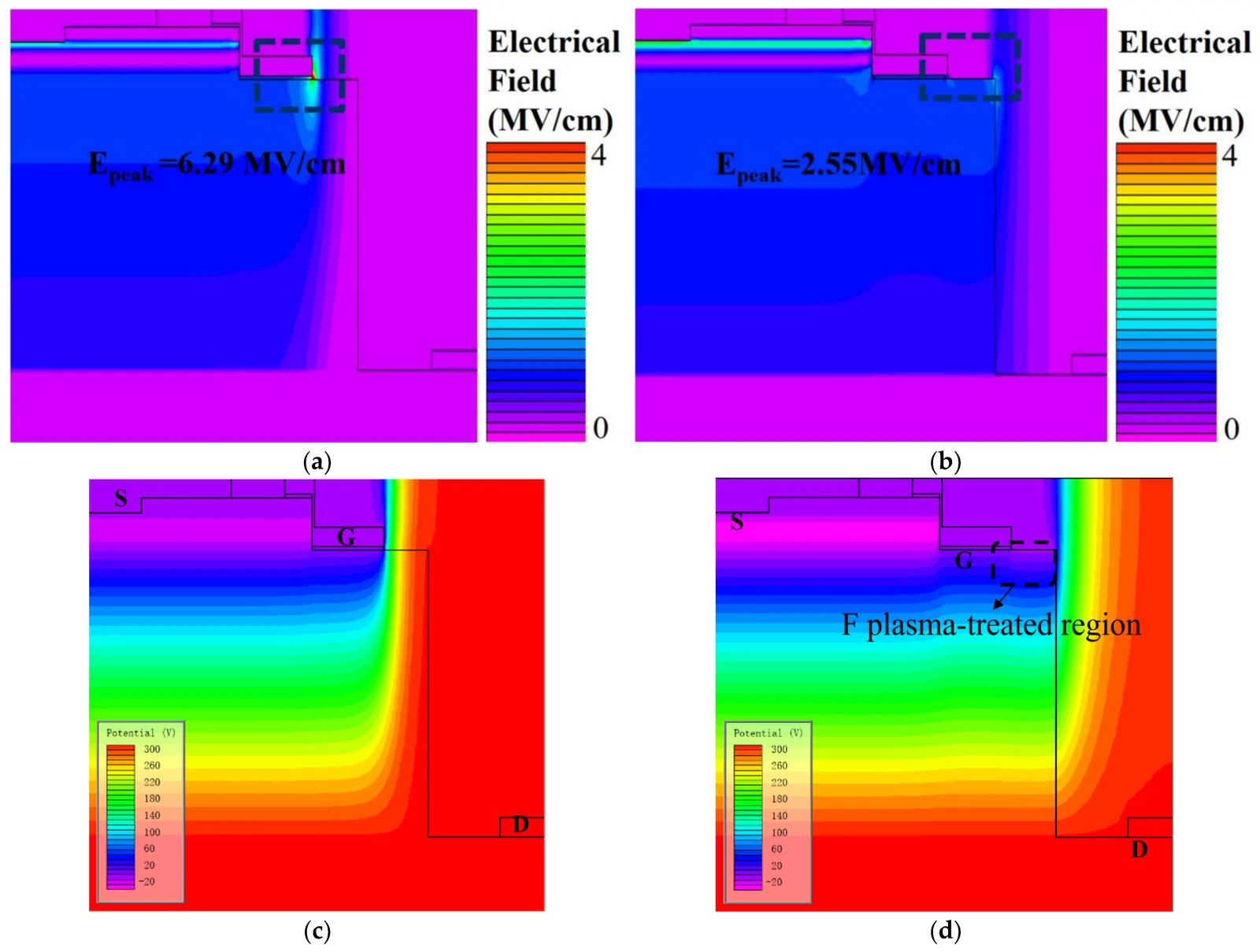
Simulation potential distribution of device (**a**) without and (**b**) with F-plasma treatment at 300 V. Simulation of electric field distribution of device (**c**) without F-plasma treatment and (**d**) with F-plasma treatment at 300 V.

The off-state characteristics of the as-fabricated quasi-vertical GaN-based trench-gate MOSFETs are plotted in Figure 3. The measured breakdown voltages (V_br_) of conventional device A, conventional device B, and the F-plasma-treated device are 170 V, 100 V, and 275 V, respectively. The degraded Vbr of device B relative to device A originates from the reduced gate-to-drain spacing and the deeply etched drift region sidewall: the elevated etching power induces more severe etching damage, rendering the device more susceptible to premature breakdown. With F-plasma treatment, the pre-breakdown leakage current is substantially suppressed, and the resulting V_br_ far exceeds those of both conventional devices A and B. This improvement stems from the fixed negative charges introduced in the F-plasma-treated region, which repel electrons and induce a depletion region. This effect serves to spread the electric field distribution and suppress the peak electric field magnitude [16,17]. Figure 3b summarizes the statistical distribution of V_br_ measured across different devices. Figure 4a,b depict the potential profiles of devices with and without F-plasma treatment. Simulations reveal that the potential within the F-plasma-treated region is significantly lowered compared with the untreated counterpart, verifying that the incorporated fixed negative charges effectively reduce the local potential and flatten the potential distribution. Figure 4c,d display the off-state electric field distribution in the reference and F-plasma-treated devices under a drain bias of 300 V. The peak electric field drops from 6.29 MV/cm to 2.55 MV/cm, and the severe electric field crowding at the trench gate is effectively mitigated. The combined reduction in gate potential and peak electric field accounts for the enhanced breakdown performance.

**Figure 5:**
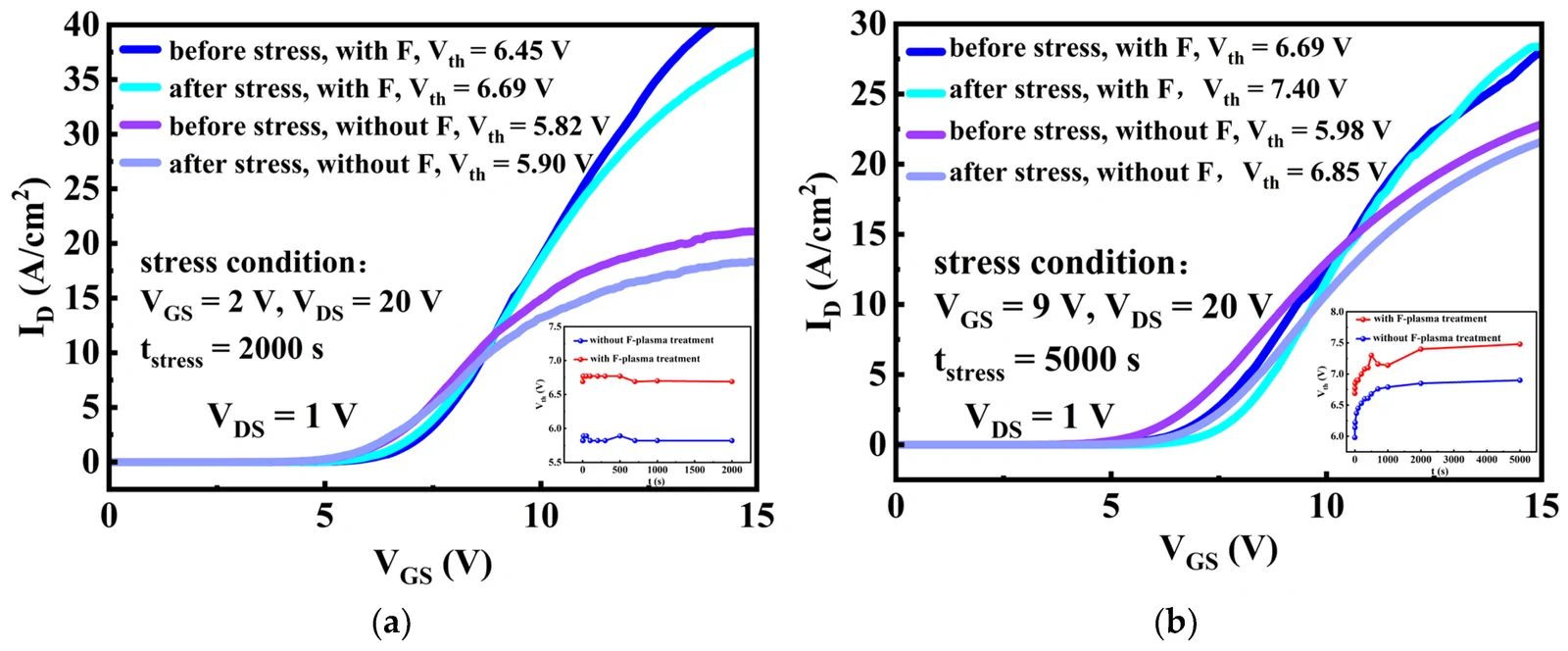
(**a**) Comparison of transfer characteristics before and after off-state stress (2000 s) for the device without and with F-plasma treatment and variation in device threshold voltage with off-state stress time for the device with and without F-plasma treatment. (**b**) Comparison of transfer characteristics before and after on-state stress (5000 s) for the device with and without F-plasma treatment and variation in device threshold voltage with on-state stress time for the device with and without F-plasma treatment.

Figure 5a compares the transfer characteristics of the untreated and F-plasma-treated devices before and after off-state stress. Off-state stress shows negligible effect on the threshold voltage of both devices. Figure 5b presents the transfer characteristics of the two devices before and after on-state stress. After 5000 s of on-state stress, the threshold voltage of the untreated device increases by 0.87 V, while that of the F-plasma-treated device rises by 0.71 V. For both devices, the threshold voltage first increases and then stabilizes with increasing stress time. A similar rise in threshold voltage with extended on-state stress time has also been reported by Zhu et al. [18]. Based on the variation trend and amplitude of the threshold voltage, F-plasma treatment introduces almost no additional effects on the device under stress conditions. This is because the F-plasma-treated region is located far from the inversion channel and does not affect the inversion layer. It can be inferred that F-plasma treatment has minimal impact on stable device operation under prolonged on-state conditions. The significant effect of on-state stress on quasi-vertical GaN-based trench gate MOSFETs is speculated to arise from the following mechanism: under high gate stress, trap states may be generated or activated at the interface between the gate dielectric and GaN. These traps capture charge carriers, thereby reducing the gate controllability of the device.

**Figure 6:**
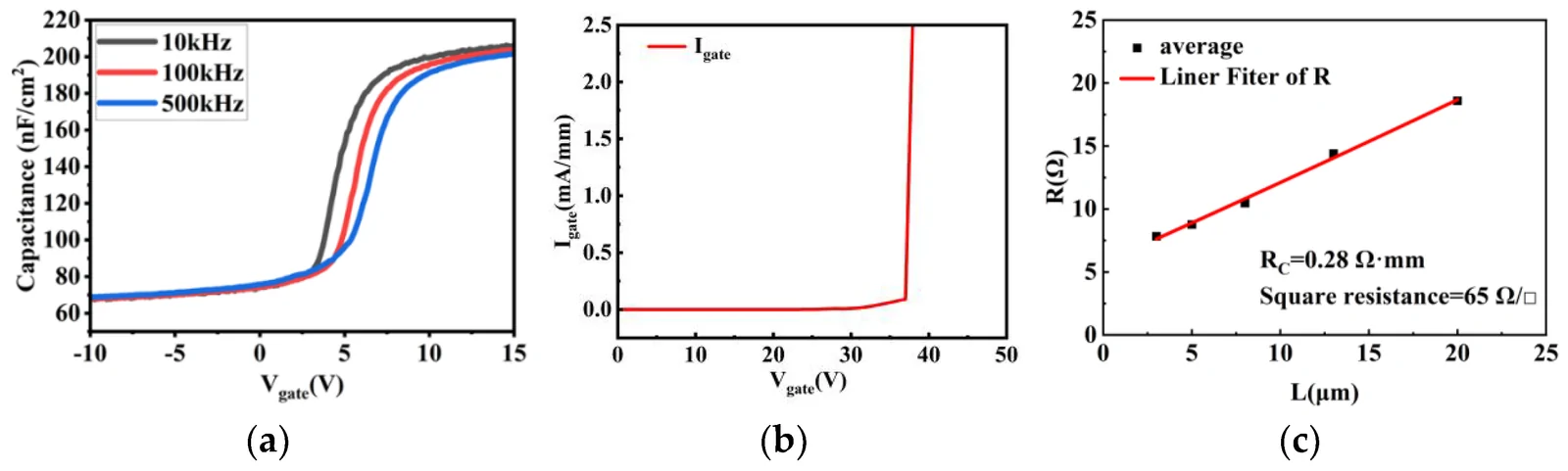
(**a**) The curve of the CV test for the device. (**b**) Breakdown curve of alumina dielectric. (**c**) TLM graphical measurement results of the device.

Figure 6a shows the results of the CV test for the device. The CV test allows for the evaluation of the device’s gate control capability and the thickness of the gate dielectric. The measured capacitance is the gate dielectric capacitance, and the gate dielectric thickness can be calculated using its theoretical dielectric constant and gate capacitance. The calculated alumina thickness from the CV test results is 41.7 nm. As can be seen from Figure 6b, the breakdown voltage of the alumina dielectric is 37 V, leading to a breakdown field strength of approximately 8.87 MV/cm. Figure 6c shows that the contact resistance R_C_ measured by TLM is 0.4 Ω·mm, and the ohmic contact resistance R_C_ measured is 0.28 Ω·mm. This result demonstrates that both the deposited dielectric film and the fabricated ohmic contact achieve favorable quality in the present experiment.

## 4. Discussion

This paper presents the fabrication of F-plasma-treated quasi-vertical GaN-based trench-gate MOSFETs and investigates the impact of F-plasma treatment on their electric field distribution and potential profiles. The results show that F-plasma treatment modulates the electric field distribution at the gate region, effectively reducing the peak electric field strength. Compared with untreated devices, the peak electric field of the treated device is reduced from 18.7 MV/cm to 4.08 MV/cm. With F-plasma treatment, the breakdown voltage of the fabricated device is successfully increased from 170 V to 275 V. Stress test results indicate that the threshold voltage of both treated and untreated devices is affected by interface charges and traps, and F-plasma treatment imposes no additional impact on the threshold voltage stability of the device. It is expected that under prolonged on-state operation, the treatment will have minimal influence on stable device performance. The foregoing results indicate that F-plasma treatment represents a promising approach worthy of investigation for both gate-controlled channel devices operating at high voltage, high frequency or high power and devices with gate oxides prone to electric field-induced degradation. The F-plasma-treated quasi-vertical GaN-based trench gate MOSFETs are expected to have a significant impact on medium- and high-voltage applications.

## Data Availability

The original contributions presented in this study are included in the article/Supplementary Materials. Further inquiries can be directed to the corresponding authors.

## Supplementary Materials

The following supporting information can be downloaded at: https://www.mdpi.com/article/10.3390/mi17091019/s1, Table S1. GaN material simulation parameter table.
