# Supplementary material for: Quasi-Vertical GaN-Based Trench Gate MOSFETs Treated with Fluorine-Plasma
Source: Micromachines (Basel). 2026 Aug 27;17(9):1019. doi: 10.3390/mi17091019 (PMC13609396; doi:10.3390/mi17091019)
Supplement: Supplementary file 1 [file micromachines-17-01019-s001.zip › micromachines-4446218-supplementary.pdf]

Parameters and models designed in simulation

S1. Parameters

Table S1. GaN material simulation parameter table.

Parameter	Value	Unit
Hole concentration coefficient of SRH	4×10^{18}	cm^{-3}
Electron concentration coefficient of SRH	4×1018	cm-3
Hole lifetime	2×10^{-9}	S
Electronic lifetime	0.7×10^{-9}	S
Hole saturation velocity	7×10^{6}	cm/s
Electron saturation velocity	1.9×10^{7}	cm/s
Hole mobility	10	$cm^2V^{-1}s^{-1}$
Electron mobility	1000	$cm^2V^{-1}s^{-1}$
Donor activation energy	17	meV
Acceptor activation energy	240	meV
Electron affinity	4.1	eV
Relative permittivity	9.5	-
Band gap (300K)	3.4	eV
Conduction band state density (300K)	1.16×10^{19}	cm^{-3}
Valence band state density (300K)	2.07×10^{18}	cm^{-3}

The thickness and doping concentration of the material used in the simulation are the same as those on page 3 of the manuscript. Since a two-dimensional (2D) device architecture is adopted in the simulation, the z-direction dimension of the gate trench (Lgrass) is set to 1 um by default in the simulation tool.

S2. Models

To meet the simulation requirements for quasi-vertical GaN MOSFETs, this manuscript establishes a model framework incorporating four pivotal physical effects: the mobility model, impact ionization model, recombination model, and incomplete ionization model.

Recombination model: Three classical models are widely adopted to characterize non-equilibrium carrier recombination: the direct recombination model, the indirect recombination model, and the Auger recombination model. In this paper, the Shockley–Read–Hall (SRH) defect-assisted recombination model is selected for recombination modeling. As a typical indirect recombination mechanism, the SRH model achieves the stepwise recombination of carriers through defect energy levels serving as intermediate states.

Carrier mobility model: In this work, a hybrid modeling strategy is employed. Through the synergistic coupling of the fldmob and conmob models, the proposed framework can dynamically identify the dominant governing factor between electric field intensity and carrier concentration, and intelligently select the optimal model combination accordingly. This dual-model architecture

more accurately captures the nonlinear dependence of carrier mobility on bias voltage in GaN devices.

Impact ionization model: In the simulation of the quasi-vertical GaN-based MOSFET presented in this manuscript, the device breakdown is identified as avalanche breakdown induced by impact ionization, and the Selberherr model is adopted as the impact ionization model for numerical calculation.

F-plasma treatment: In this manuscript, the effect of fluorine plasma treatment is numerically emulated by introducing fixed negative charges, which facilitates the systematic investigation of quasi-vertical GaN-based MOSFETs subject to fluorine plasma treatment.
